# Supplementary figures and images for: Head and Body Dyskinesia During Gait in Tactical Athletes With Vestibular Deficit Following Concussion
Source: Front Sports Act Living. 2021 Aug 10;3:703982. doi: 10.3389/fspor.2021.703982 (PMC8384176; doi:10.3389/fspor.2021.703982)

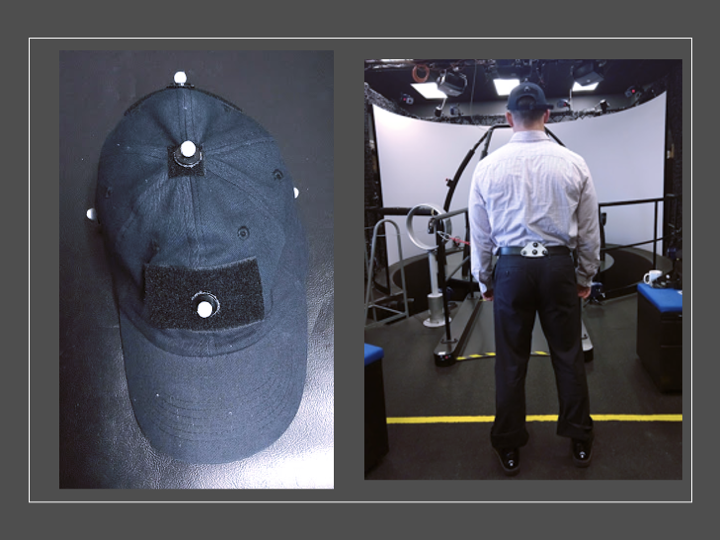

Supplement: Supplementary Figure 1 — Photo-reflective marker placement for three-dimensional motion capture of the head and pelvis during walking. [file Image_1.TIFF]
